# Efficacy and Safety of NFL-101 as a Smoking Cessation Therapy: A Randomized Phase II Clinical Trial CESTO2

**DOI:** 10.1093/ntr/ntaf181

**Published:** 2025-08-30

**Authors:** Claire Lafay-Chebassier, Pierre-Olivier Girodet, Fabrice Laine, Jean-Sebastien Allain, Gisele Pickering, Mathilde Latreille, Anastasia Demina, Hugues Chevassus, Isabelle Ingrand, Eric Tartour, Nadine Benhamouda, Marie-Laure Fraisse, Liliya Chamitava, Yannick Plétan, Julie Balland, Yves Donazzolo, Bruno Lafont

**Affiliations:** INSERM CIC 1402, Université de Poitiers, CHU de Poitiers, Poitiers, France; Laboratoire de Neurosciences Expérimentales et Cliniques, INSERM U-1084, Université de Poitiers, Poitiers, France; Service de Pharmacologie Clinique et Vigilances, CHU de Poitiers, Poitiers, France; CIC1401, U1045, Pharmacology Department University of Bordeaux , Bordeaux, France; Institut national de la santé et de la recherche médicale CIC1401, U1045, Bordeaux, France; Service de Pharmacologie médicale Centre Hospitalier Universitaire de Bordeaux Pessac, Pessac, France; Centre d’Investigation Clinique CHU-Rennes (CIC 1414), CHU Rennes, Institut National de la Santé et de la Recherche Médicale, Inserm, Rennes, France; Liver Unit, CHU Pontchaillou, Rennes, France; Department of Internal Medicine, Centre Hospitalier Bretagne Sud, Lorient, France; Plateforme d'Investigation Clinique/CIC INSERM U-1405, University Hospital CHU, Clermont-Ferrand, France; Eurofins Optimed, Giéres, France; Addiction Medicine Department, Dijon Bourgogne University Hospital, Dijon, France; INSERM U-1093, CAPS, Université de Bourgogne, UFR STAPS, Dijon, France; Clinical Investigation Center, Montpellier University Hospital, INSERM, CIC1411, Montpellier, France; Registre des Cancers Poitou-Charentes, Université de Poitiers, Poitiers, France; Service d'immunologie biologique, plateforme d'immunomonitoring, Hôpital européen Georges-Pompidou, AP-HP, Paris, France; Université Paris-Descartes, Sorbonne-Paris-Cité, INSERM, PARCC, Paris, France; Service d'immunologie biologique, plateforme d'immunomonitoring, Hôpital européen Georges-Pompidou, AP-HP, Paris, France; Université Paris-Descartes, Sorbonne-Paris-Cité, INSERM, PARCC, Paris, France; NFL Biosciences, Castelnau-Le-Lez, France; Valos S.r.L., Genova, Italy; NFL Biosciences, Castelnau-Le-Lez, France; Solid Drug Development, Geneva, Switzerland; Eurofins Optimed, Giéres, France; NFL Biosciences, Castelnau-Le-Lez, France

## Abstract

**Introduction:**

Tobacco addiction remains a major public health challenge. Existing smoking cessation treatments require prolonged daily use with potentially poor adherence and reduced efficacy.

**Methods:**

The phase II study was a multicenter, randomized, double-blind, placebo-controlled trial with a 1-year follow-up to assess the efficacy, safety, and immunogenicity of NFL-101 as a potential aid for smoking cessation. A total of 318 adult daily smokers were randomized to receive subcutaneous injections of NFL-101-100 μg, NFL-101-200 μg, or placebo on day 1 and day 8. The primary outcome was 6-week post-quit 28-day continuous abstinence (CA, day 15–day 43), validated by exhaled CO.

**Results:**

CO verified 6-week post-quit CA was: NFL-101-100 μg: 31/108 (28.7%), 200 μg: 23/109 (21.1%), and placebo: 18/101 (17.8%), NFL-101-100 μg vs placebo, RR = 1.61, *p* = .063, and 200 μg vs placebo RR = 1.18, *p* = .5492. CA, when urinary cotinine was used, was: 26/108 (24.1%) for NFL-101-100 μg, 18/109 (16.5%) for 200 μg, and 13/101 (12.9%) for placebo. NFL-101-100 μg vs placebo showed an RR = 1.87, 95% confidence interval (CI): 1.02 to 3.44, *p* = .0378 and 200 μg vs placebo was RR = 1.28, 95%CI: 0.66 to 2.48, *p* = .4572. If individuals who used NRTs and/or e-cigarettes were classified as non-abstinent, then 29/108 (26.9%) were abstainers for NFL-101-100 μg and 14/101 (13.9%) for placebo (*p* = .0203). NFL-101-100 μg RR remained stable between 28 days and 12 months. At day 43, NFL-101-100 μg reduced craving (*p* < .05), with no significant difference for withdrawal symptoms. Abstainers experienced greater increases in anti-NFL-101-IgG concentrations compared to non-abstainers (*p* < .009). NFL-101 was well-tolerated.

**Conclusions:**

Although the prespecified primary endpoint was not statistically significant, if the primary outcome had been defined as nicotine abstinence, the results would have reached statistical significance. Efficacy, craving reduction, and minimal dosing regimen of NFL-101-100 μg support its potential as a promising smoking cessation therapy.

**Implications:**

In this multicenter randomized clinical trial that included 318 smokers, effect sizes between groups were sufficiently large to suggest a meaningful clinical effect. NFL-101 at a dose of 100 μg increased 6-week post-quit 28-day continuous smoking abstinence that was confirmed by urinary cotinine concentrations and reduced craving, suggesting psychological benefits that could mitigate relapse risks. Abstainers experienced a significant increase in anti-NFL-101 IgG concentrations compared to those who continued to smoke.

Findings from the present study offer support for an entirely new category of treatment that acts through immune modulation. Additional strengths include a subcutaneous route of administration in the form of two injections spaced a week apart (thus, enhances treatment adherence) and has demonstrated a safe profile with minimal adverse effects. These results support a follow-up phase III clinical trial.

## Introduction

According to the World Health Organization (WHO), tobacco is one of the world’s largest preventable causes of premature death, accounting for more than 8 million deaths and costing the global economy US $1.4 trillion each year.^1^ Smoking cessation is beneficial at any age as it improves health status and quality of life.^2^ Hence, reducing tobacco use has been identified as a major focus in global efforts to achieve the Sustainable Development Goals target to reduce premature deaths from noncommunicable diseases by one-third by 2030.^3^

Current best practice recommendations are to treat tobacco dependence using a combination of behavioral counseling and pharmacotherapy using one of several medications: nicotine replacement therapy (NRT), varenicline, bupropion, or cytisine. These medications have varying success rates in helping individuals achieve abstinence, but all have side effects (some significant) and require administration between 1 and 3 times per day throughout treatment. Adherence to pharmacotherapy tends to be low.^4^^,^^5^ For example, varenicline is intended to be taken for at least 12 weeks, yet self-reported data show that only 33% of smokers were still taking it 12 weeks after their quit date.^6^ These data highlight the need for novel treatments to expand the options available for smoking cessation especially therapies that can improve treatment adherence.

NFL-101 is a standardized sterile aqueous extract of tobacco leaves that is subcutaneously administered twice, 1 week apart, to help smokers quit. Its origin traces back to an allergenic extract previously used to desensitize tobacco factory workers with tobacco leaf allergies, which was authorized until 2004.^7^ The theoretical basis for using NFL-101 is to activate components of the immune system and create an aversive or at least non-rewarding response to nicotine and other components of tobacco smoke. The goal is that two injections of NFL-101 will make tobacco smoking less enjoyable and/or desirable and thus lead to abstinence. NFL-101 induced a preferential and rapid activation of human NK cells *in vitro*, with an anti-NFL-101 IgG humoral immune response, and restored normal brain glucose metabolism in mice that had been exposed to cigarette smoke.^8^

In the phase I CESTO clinical trial, ^9^ all volunteers reported significant changes in their response to smoking after NFL-101.^10^ Examples of verbal reports from participants who received NFL included: “less desire to smoke,” “disgust with cigarettes,” “disgust when smoking,” “the impossibility of smoking the cigarette until the end,” and “an absence of craving.” The results of the phase I CESTO clinical trial demonstrated that NFL-101 was safe with adverse effects comparable to those observed in the placebo group.^10^ The phase IIa PRECESTO trial demonstrated clear efficacy of NFL-101 over placebo in reducing the positive reinforcing effects of cigarettes in nontreatment-seeking smokers. Compared to placebo, NFL-101 significantly reduced participants’ cigarette smoking satisfaction over a 28-day period.^11^^,^^12^ The safety profile was confirmed in this second study.^12^

In the present study, we present the results of a phase II multicenter, randomized, double-blind, placebo-controlled trial (CESTO 2) conducted to evaluate the efficacy of NFL-101 as smoking cessation therapy, to study immunogenicity as a possible mechanism of action and to confirm safety in a larger population.^13^^,^^14^

## Methods

### Setting

CESTO 2 study was a multicenter, randomized, double-blind, three parallel-group placebo-controlled trial. This study was conducted in nine centers in France, in accordance with the Declaration of Helsinki, Good Clinical Practice guidelines, ICH guidelines, and relevant French laws. The study was approved by the Ethical Committee of 06/09/2019 (n° 170 914) and registered with EudraCT n° 2017-002897-38 within the French clinical trial public registry on the ANSM website (http://ansm.santé.fr/) and on ClinicalTrials.gov (NCT04571216). All participants provided written informed consent and were randomized from March 2022 through May 2023 and follow-up was completed by May 2024.

### Participants

Eligible adult male or female participants (aged 18–70 years) in good physical and mental health with an Eastern Cooperative Oncology Group and World Health Organization (ECOG/WHO) performance status between 0 and 1, who smoked at least 11 cigarettes per day, had a Fagerström Test for Nicotine Dependence (FTND) score and were willing to quit smoking were recruited. Exclusion criteria included alcohol or drug abuse (including cannabis) in the past year, psychiatric history (adequately treated depression was accepted), abnormal electrocardiogram (ECG) recording on a 12-lead ECG at screening, and positive prick test at screening for NFL-101. Women of childbearing potential had to have a negative pregnancy test at screening and then agree to use highly effective methods of contraception for the duration of the trial.

Additional exclusionary criteria included: any current active infectious disease, concomitant use of treatment known to interfere with the immune system, uncontrolled diabetes, prior exposure to a vaccine up to 30 days before NFL-101 administration, concomitant use (and within previous 60 days) of any smoking cessation therapy (including electronic cigarettes and alternative methods such as hypnosis or acupuncture), pregnancy, and breastfeeding.

### Randomization and Interventions

Participants were randomly assigned in a 1:1:1 ratio to receive either NFL-101 100 μg, 200 μg, or placebo administered as two subcutaneous injections (one in each arm) on day 1 and day 8. Optional administrations were offered to individuals who were not abstinent on day 99 and day 196. Randomization was stratified by assigned sex at birth (male or female) and FTND score (three classes depending on score values, 1–3, 4–6, and 7–10 corresponding to low, medium, and high dependence, respectively) using a variable block size multiple of 3 in order to achieve a 1:1:1 ratio between the two active doses and placebo. The randomization list was generated by Eurofins Optimed with validated SAS program.

Investigators, participants, and sponsor’s clinical trial team members were blind to the randomization assignments. To maintain the study blind, the syringes were wrapped with aluminum foil and injections were administered by nurses who had no other role in the study; participants had their backs turned during treatment administration.

### Assessments

Participants were seen on site on days 1 (at least 15 days after screening), 8, 15, 29, 43, 85 (M3), 186 (M6), 274 (M9), and 365 (M12); optional visits were included on days 99, 113, 196, and 210 for individuals who received treatments on D99 and/or D196.

Data collected during these visits included clinical examination (vital signs such as heart rate and blood pressure), history of past tobacco use, specific treatment presentation, counseling, date and type of concomitant therapies for smoking cessation (NRT and e-cigarettes), urinary cotinine and exhaled carbon monoxide (CO) concentrations, the Minnesota Nicotine Withdrawal Scale (MNWS) questionnaire, the Tobacco Craving Questionnaire—12 items (FTCQ-12), anti-NFL-101 IgG concentrations, pregnancy test, urine drug screens, concomitant medications, and adverse events (AEs). On screening visit and day 365, blood and urine samples were collected to measure key hepatic, renal, hematologic, and electrolyte markers and non-fasting glycemia. The use of NRTs was not supervised and left at the discretion of individual centers after day 15.

### Outcome Measures

The primary endpoint was defined as continuous abstinence (CA) from smoking for 28 days or more from D15 to D43, as assessed by self-reported abstinence from smoking that was confirmed by expired CO concentration of 10 ppm or less. Secondary endpoints included confirmed CA for 3, 6, 9, and 12 months from D15. Other endpoints were the perceived effect of treatment, immunogenicity, and safety. The perceived effect of treatment was assessed by the MNWS questionnaire assessing withdrawal symptoms including craving, and the FTCQ-12 evaluating craving and its various components (emotionality, expectancy, compulsivity, and purposefulness). Immunogenicity was assessed by specific anti-NFL-101 IgG determination by ELISA in serum.

Safety was assessed via the monitoring of AEs and treatment-emergent adverse events (TEAEs), severity or dose-limiting toxicity, laboratory data, vital signs, 12-lead ECG, and physical examination.

### Statistical Analysis

The required sample size was estimated for a 2-group ×2 test independently comparing each of the two doses to placebo at 0.0125 one-sided significance level, the alternative hypothesis to be detected being a success rate of 29% for the best dose, assuming a 10% success rate under placebo. The approach of randomizing 318 participants in 1:1:1 proportion to each of the three groups provided more than 90% power for the two doses to reject the null hypothesis if not different from placebo, if the alternative was true. An observed outcome of 10% success rate with placebo and more than 21% with the best dose allowed rejection of the null hypothesis. Analyses on the efficacy endpoints were performed on the intent-to-treat set with all randomized participants who received at least the first treatment administration. Individuals who received optional doses on D99 and/or D196 were non-abstinent at D85 (M3) or D186 (M6) and therefore could not be continuously abstinent from D15 during the periods D85 (M3) or D186 (M6).

Continuous numerical variables were summarized using the following descriptive statistics: *n*, mean, standard deviation, 95% confidence interval (CI) for the mean, minimum, maximum, and risk ratios (RRs); categorical variables were summarized descriptively with frequencies and percentages. CA was analyzed inferentially by means of Pearson’s chi-square (χ^2^) tests to assess the proportion of participants who succeeded (Yes/No) in achieving abstinence. If the distribution of participants across levels of success (ie, Yes or No) and the visits under scrutiny were 5 or less in at least one case, then Fisher’s exact test was used. Separate comparisons were made for each of the two active dose groups vs placebo.

Exploratory post hoc analyses were conducted to confirm continuous abstinence by high-performance liquid chromatography (HPLC) measures of urinary cotinine concentrations (<0.05 μg/mL) as the biomarker instead of exhaled CO and to also assess nicotine abstinence by classifying any individual who reported using NRTs or e-cigarettes as being “non-abstinent” to more accurately assess the treatment’s intrinsic effect as per FDA recommendations.^15^

Three-way ANOVA models were used to analyze data on the perceived effect of the treatment. The models included randomized treatment (100 μg dose vs placebo, and 200 μg dose vs placebo), sex, and Fagerström score at baseline covariates as fixed effects, and the change from baseline to the post-baseline visits as the dependent variable. Mixed models for repeated measure (MMRMs) were employed for each of the perceived effect items. The absolute change from baseline to the post-baseline visits was the dependent variable and included the following covariates: randomized treatment, sex, Fagerström score at baseline, and at each study visit. A repeated measure effect for each participant was included for the visit factor and an unstructured covariance matrix was applied to capture the within-subject correlations across repeated measures.

Immunogenicity was also analyzed with an MMRM. The model included the fixed effects of CA confirmed by urinary cotinine over 4 weeks (D15–D43) or over 12 months (D15–M12), visit and abstinence by visit interaction, and baseline anti-NFL-101 IgG as a covariate. An unstructured covariance structure was used to model the within-subject error. The Kenward–Roger approximation was used to estimate the denominator degrees of freedom and the model was computed using restricted maximum-likelihood.

For primary analysis, α was set to 0.025, one-sided. All other statistical tests were carried out at a significant level of 0.05, two-tailed. All statistical analyses were performed independently using SAS version 9.4 (SAS Institute Inc, Cary, NC, USA).

Protocol writing, database, and data monitoring were conducted by Eurofins Optimed, which conducted routine monitoring to provide centralized data collection from investigators at each participating sites. Valos was in charge of data management and clinical study report writing.

## Results

### Participants

**Figure 1:**
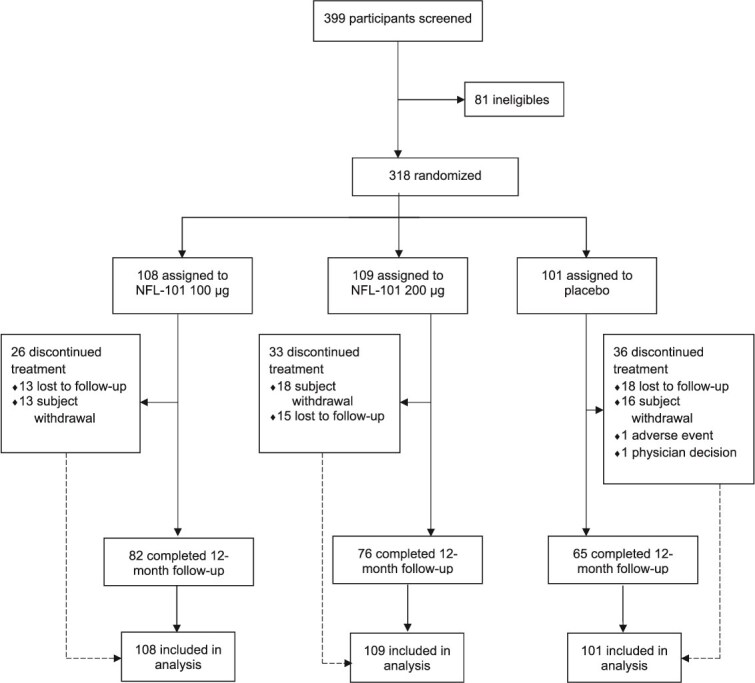
Consort flow diagram.

Of the 399 individuals who were screened, 321 were eligible; finally, 318 received at least one treatment administration: 100 μg NFL-101 (*n* = 108), 200 μg NFL-101 (*n* = 109), or placebo (*n* = 101) (Figure 1). A total of 41%, 34%, and 49% of participants from the 100 μg NFL-101, 200 μg NFL-101, and placebo groups, respectively, received three or four treatments. Ninety-five (30%) participants admitted to have taken NRT/e-cigarettes between D15 and D43: 28 (26%) in the 100 μg dose group, 32 (29.3%) in the 200 μg dose group, and 35 (34.6%) in the placebo group. Overall, the 12-month follow-up assessment was completed by 82 (76%) participants in the 100 μg NFL-101 group, 76 (70%) in the 200 μg NFL-101 group, and 65 (64%) in the placebo group (Figure 1). The recruitment flow chart and reasons for discontinuation are presented in Figure 1.

Baseline characteristics of study participants were comparable in all groups of treatment (Table 1). Mean age was 49.6 years (18–70), and sex distribution was roughly equal (51.6% male, 48.4% female). The average number of cigarettes smoked was 18.9 per day and the FTND score averaged 5.5 across all groups.

**Table 1:** Baseline Characteristics of Study Participants by Treatment Groups

	**100 μg NFL-101** **(*n* = 108)**	**200 μg NFL-101** **(*n* = 109)**	**Placebo** **(*n* = 101)**
Age, mean (min-max), (years)	49.5 (23–68)	49.1 (18–70)	50.3 (22–69)
Gender, no. (%)			
Male	56 (51.9)	59 (54.1)	49 (48.5)
Female	52 (48.1)	50 (45.9)	52 (51.5)
**Tobacco use, mean (SD)**			
Duration of smoking, years	33.9 (10.7)	33.0 (11.2)	34.0 (10.6)
Number of cigarettes per day	18.2 (6.3)	19.8 (7.0)	18.5 (5.2)
Exhaled carbon monoxide, ppm	22.2 (11.3)	22.7 (11.8)	23.3 (12.5)
Urinary cotinine, μg/mL	1 464 (791)	1 562 (701)	1 474 (570)
Fagerström Test for Nicotine Dependence score	5.5 (1.7)	5.5 (1.7)	5.7 (1.5)
French Tobacco Craving Questionnaire-12 items score	2.7 (0.8)	2.7 (0.7)	2.5 (0.6)
**Quitting history**			
Prior quit attempts, mean (SD)	3.2 (2.5)	3.7 (3.5)	3.8 (3)
Prior cessation therapy used, no. (%)			
Oral nicotine substitute	16 (14.8)	29 (26.6)	27 (27.7)
Patch	57 (52.8)	48 (44)	58 (57.4)
Electronic cigarette	33 (30.6)	32 (29.4)	39 (38.6)
Hypnosis	33 (30.6)	35 (32.1)	44 (43.6)
Acupuncture	15 (13.9)	20 (18.3)	20 (19.8)
Varenicline	20 (18.5)	13 (11.9)	17 (16.8)
Bupropion	9 (8.3)	8 (7.3)	4 (4)
Willpower only	47 (43.5)	51 (46.8)	37 (36.6)

### Efficacy

#### Smoking Outcomes

No statistically significant difference between NFL-101 and placebo was observed in the proportion of individuals who remained abstinent from D15 to D43 as confirmed by exhaled CO concentration (≤10 ppm). In the 100 μg dose group, abstinent rates were 31/108 (28.7%) vs 18/101 (17.8%) in the placebo group (*p* = .063; RR = 1.61) (Table 2). For the 200 μg dose group, abstinent rates were 23/109 (21.1%) vs 18/101 (17.8%) in the placebo group (*p* = .5492; RR = 1.18) (Table 2).

**Table 2:** Biochemically Confirmed Continuous Smoking Abstinence by Treatment Group

		**No. (%)**			**100 μg NFL-101 vs placebo**		**200 μg NFL-101 vs placebo**	
		**100 μg NFL-101**	**200 μg NFL-101**	**Placebo**	**Relative risk (95% CI)**	***p*-value**	**Relative risk (95% CI)**	***p*-value**
		**(*n* = 108)**	**(*n* = 109)**	**(*n* = 101)**				
**Primary outcomes**								
4-week (D15–D43) confirmed by^*^	Exhaled CO (<10 ppm)	31 (28.7)	23 (21.1)	18 (17.8)	1.61 (0.96–2.69)	.0635	1.18 (0.68–2.06)	0.5492
	Urinary cotinine (<0.05 μg/mL)	26 (24.1)	18 (16.5)	13 (12.9)	1.87 (1.02–3.44)	**.0378**	1.28 (0.66–2.48)	0.4572
**Secondary outcomes**								
3-month (D15–D85) confirmed by^*^	Exhaled CO (<10 ppm)	23 (21.3)	17 (15.6)	13 (12.9)	1.65 (0.89–3.09)	.1070	1.21 (0.62–2.37)	.5729
	Urinary cotinine (<0.05 μg/mL)	20 (18.5)	13 (11.9)	11 (10.9)	1.70 (0.86–3.37)	.1211	1.10 (0.51–2.33)	.8137
6-month (D15–D85) confirmed by^*^	Exhaled CO (<10 ppm)	18 (16.7)	16 (14.7)	10 (9.9)	1.68 (0.82–3.47)	.1513	1.48 (0.71–3.11)	.2936
	Urinary cotinine (<0.05 μg/mL)	15 (13.9)	12 (11.0)	8 (7.9)	1.75 (0.78–3.96)	.1683	1.39 (0.59–3.26)	.4462
9-month (D15–D274) confirmed by^*^	Exhaled CO (<10 ppm)	18 (16.7)	12 (11.0)	10 (9.9)	1.68 (0.82–3.47)	.1513	1.11 (0.50–2.46)	.7933
	Urinary cotinine (<0.05 μg/mL)s	15 (13.9)	9 (8.3)	8 (7.9)	1.75 (0.78–3.96)	.1683	1.04 (0.42–2.60)	.9289
12-month (D15–D365) confirmed by^*^	Exhaled CO (<10 ppm)	17 (15.7)	11 (10.1)	10 (9.9)	1.59 (0.76–3.31)	.2085	1.02 (0.45–2.30)	.9633
	Urinary cotinine (<0.05 μg/mL)	14 (13.0)	9 (8.3)	7 (6.9)	1.87 (0.79–4.45)	.1472	1.19 (0.46–3.08)	.7174

^*^Post hoc analysis for urinary cotinine and exhaled CO. In bold: *p*-value<0.05.

The following abstinence rates were apparent when urinary cotinine concentrations were used: 26/108 (24.1%) for NFL-101-100 μg, 18/109 (16.5%) for NFL-101-200 μg, and 13/101 (12.9%) for placebo. NFL-101-100 μg vs placebo showed RR = 1.87 (95%CI: 1.02 to 3.44; *p* = .0378) and NFL-101-200 μg vs placebo showed RR = 1.28 (95%CI: 0.66 to 2.48; *p* = .4572).

Abstinence from D15 to D43 was observed in 26.9% of the NFL-101-100 μg group versus 13.9% in placebo (*p* = .020) when classifying any individual who used NRTs or e-cigarettes as being non-abstinent. Among abstinent participants, two (6.5%), two (8.7%), and four (22.2%) reported using NRTs/e-cigarette, respectively, in the 100 μg dose, 200 μg dose, and the placebo groups.

Rates of CA from smoking at 3, 6, 9, and 12 months following NFL-101 administration did not differ statistically from those in the placebo group, except from D15 to M3 (20.4% vs 9.9% in placebo, *p* = .035) when participants using NRTs or e-cigarettes were considered non-abstinent. From D15 to M12, none of the continuous abstinent participants reported using NRTs/e-cigarette in any group. Risk ratios remained stable over time between CA from D15 to D43 and from D15 to M12 when CA was confirmed by exhaled CO or by urinary cotinine (Table 2).

#### Withdrawal Symptoms and Tobacco Craving

**Figure 2:**
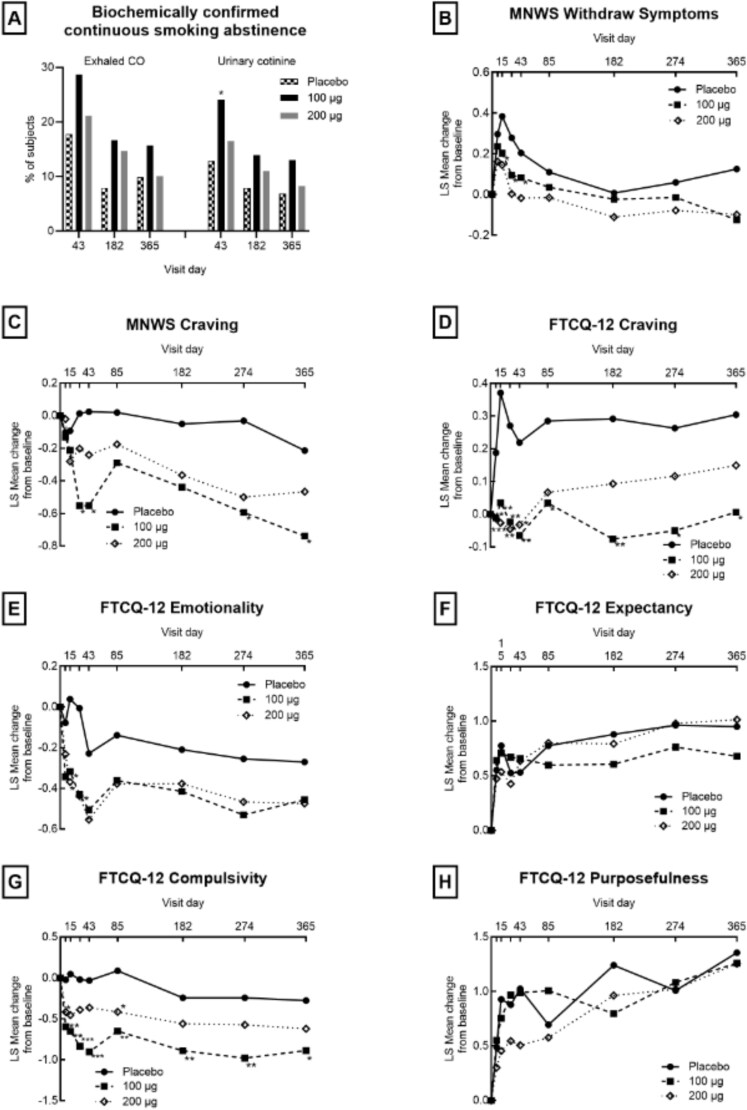
Effects of NFL-101 on continuous smoking abstinence, withdrawal symptoms, and tobacco craving. ANOVA ^*^p < .05; ^**^p < .01; ^***^p < .001.

NFL-101-100 μg did not alleviate withdrawal symptoms (Figure 2). However, an ANOVA analysis on MNWS scores revealed a significant reduction in craving at D29 (*p* = .010) and D43 (*p* = .017). The MMRM analysis confirmed this reduction at D29 (*p* = .034) and D43 (*p* = .060). Results from the FTCQ-12 revealed that NFL-101 considerably reduced craving (*p* = .002, .007, .010, .033, .004, .025, and .027 at D15, 29, 43, 85, 182, 274, and 365, respectively), emotionality (smoking’s perceived ability to improve mood or mental state) (*p* = .03 and .01 at D15 and 29, respectively), and compulsivity (urgency and difficulty in resisting the urge to smoke associated with smoking) (*p* = .007, .002, .0003, <.0001, .002, .008, .006, and .022 at D8, 15, 29, 43, 85, 182, 274, and 365, respectively), with sustained effects throughout the study as shown in Figure 2. The MMRM analysis confirmed the results on craving (*p* = .023, .050, and .037 at D15, 43, and 182, respectively), emotionality (*p* = .039, .008, and .031 at D15, 29, and 43, respectively), and compulsivity (*p* = .016 and 0.008 at D29 and 43, respectively). No effect was observed on the expectancy (anticipation of the smoking experience being pleasant or unpleasant) and purposefulness (readiness to smoke based on the situation and ability to resist) components (Figure 2).

#### Immunogenicity

At baseline, mean anti-NFL-101 IgG concentrations were similar in all groups with 8.8, 8.8, and 8.5 μg/mL in the 100 and 200 μg doses of NFL-101 and placebo groups, respectively. The increase in anti-NFL-101 IgG levels was significantly higher for the 200 μg group as compared to the 100 μg group (*p* < .02), and for each of the two active dose groups as compared to placebo (*p* < .005) (Supplementary Table SS2). Regardless of the treatment arm, participants who remained abstinent over the 4-week and 12-month periods experienced a greater increase in anti-NFL-101 IgG concentrations compared to those who continued to smoke (*p* < .009). In the 200 μg group, the differences in IgG change from baseline between abstinent and non-abstinent individuals were statistically significant across all timepoints from D29 to D182 (*p* < .02), and in the 100 μg group statistical significance was observed at D29 for the 4-week CA period and from D29 to D43 for the 12-month period. (Supplementary Table SS3).

A logistic regression model was used to estimate the odds ratios and confidence intervals of the relationships between CA for 4 weeks or 12 months, and IgG change from baseline. The probability of being CA at 12 months increased by 45%–53% in the 100 μg dose group and 23%–26% in the 200 μg dose group for each 1 μg/mL increase in IgG compared to non-abstinent. The association between CA status and IgG increase was weaker at 4 weeks compared to 12 months. In contrast, no significant differences in IgG concentrations were observed between abstinent and non-abstinent individuals in the placebo group (Supplementary Table SS3).

#### Safety

Among the 318 participants included in the safety population, a total of 196 experienced at least one AE: 74 (38%), 64 (33%), and 58 (30%) in the 100 μg, 200 μg dose, and the placebo groups, respectively. In 135 participants, AEs were considered treatment-emergent (50, 46, and 39 participants in the 100 μg, 200 μg, and placebo dose groups, respectively). Six participants experienced a serious AE, but none were considered treatment-related (one bronchial carcinoma in the 100 μg group; one breast cancer, one lung adenocarcinoma, and one vascular stent stenosis in the 200 μg group; one metastatic lung cancer and one chest pain in the placebo group). In all groups, 31.4% of TEAEs were grade 1, 17.3% of grade 2, and 0.3% of grade 3.

Supplementary Table SS1 provides an overview of the TEAEs with a System Organ Class (SOC) incidence of at least 10% of participants in either group in the 318 participants of the safety population. These were mainly infections that were equally distributed among the placebo and NFL-101 groups, systemic or local reactions and nervous system disorders (headache, dizziness) that were more prevalent in the NFL-101 groups. Psychiatric (eg, anxiety, insomnia) and gastrointestinal (nausea) disorders were evenly distributed across all groups. Overall NFL-101 was well-tolerated.

## Discussion

This phase II multicenter, randomized, double-blind, placebo-controlled clinical trial assessed the efficacy, safety, and immunogenicity of a novel drug, NFL-101, for smoking cessation.

The primary endpoint of CA, based on self-reported abstinence and exhaled CO measurement, did not achieve statistical significance. However, using a more precise and reliable biomarker, urinary cotinine concentrations, CA in the 100 μg dose of NFL-101 would have been significantly increased from D15 to D43. Unlike exhaled CO, HPLC urinary cotinine testing provides greater sensitivity and specificity, remains unaffected by respiratory status or environmental pollution, and detects nicotine exposure over 2–3 days rather than just 12 h.^16^ NFL-101 also significantly reduced craving, and two of its four factors: emotionality and compulsivity associated with smoking; these results remained robust throughout the study. In addition, when classifying NRT and e-cigarette users as non-abstinent, exhaled CO analysis revealed a significant increase in CA from D15 to D43 compared to placebo. This classification follows FDA guidance, which advises considering participants using non-study nicotine or smoking cessation products as non-abstinent when assessing an investigational drug’s therapeutic effect.^15^

NFL-101 abstinence rates are comparable to current treatments as reported in Cochrane reviews,^17^^,^^18^ the abstinence RR being 1.55 (95%CI: 1.49 to 1.61) for any form of NRT and 2.24 (95%CI: 2.06 to 2.43) for varenicline compared to placebo. In addition, a major advantage of NFL-101 is its dosing regimen as it is injected twice, separated by 1 week, and thereby eliminating the need for daily or multiple times a day dosing. Such sustained effects of any medication are well known to enhance treatment adherence. This factor is especially important in smoking cessation, given the low compliance rates reported in long-term studies of smoking cessation. In a meta-analysis of randomized controlled trials, adherence to NRT among participants was 61% compared to 26% among participants of population-based studies and adherence to NRT has been shown to double the success rate of smoking cessation.^19^ Medication non-adherence may also explain at least part of the discrepancy between the efficacy observed in tightly managed clinical trials and real-world clinical practice where nonadherence is seen at higher rates.^4^

In addition to its ability to facilitate abstinence, NFL-101 reduces craving, and more precisely the emotional and compulsive aspects of smoking, that were sustained throughout the study. This suggests that NFL-101 may temper important triggers of relapse. This is an important finding, as craving and emotional triggers are known major barriers to successful long-term cessation.^20^

Immunogenicity results revealed a dose-dependent increase in anti-NFL-101 IgG concentrations that were absent in the placebo group, suggesting that NFL-101 elicits an inherent immunogenic response. The efficacy of NFL-101 was not directly correlated with IgG concentrations. However, this is not uncommon as many vaccines and some immunomodulators exhibit a bell-shaped dose–response curve where higher doses lead to decreased efficacy.^21^^,^^22^ Regardless of the treatment arm, participants who remained abstinent over the 4-week and 12-month periods experienced a significant increase in anti-NFL-101 IgG concentrations compared to those who continued to smoke. These findings suggest that there is a potential association between the increase in anti-NFL-101 IgG following NFL-101 injection and the likelihood of sustaining CA over a 1-year observation period. Finally, NFL-101 appears to be well-tolerated as the only AE specific to NFL-101 was mild injection site pain, because of its subcutaneous administration, which distinguishes it from orally administered treatments.

## Limitations

The present study has three main limitations: (1) the CO testing to confirm continuous abstinence was used for the primary outcome whereas cotinine concentrations would have been more robust. The decision to use CO was made because the results were available immediately, compared to a delay of 2 months to receive the urinary cotinine results; (2) the primary outcome was evaluated only at 6 weeks and will need to be confirmed at 6 and 12 months, which were assessed only as secondary endpoints in the present study; (3) the concomitant use of NRTs was not supervised and was left at the discretion of individual centers after day 15. Future studies should either systematically integrate NRTs use from day 1 for a 12-week period or avoid recommending concurrent use altogether.

## Conclusion

The results of this multicenter, double-blind, placebo-controlled CESTO 2 study confirm that NFL-101, a sterile aqueous extract of tobacco leaves, is a promising novel treatment for individuals to achieve abstinence from tobacco. NFL-101 shows a potential to decrease the rate of continuous smoking abstinence over 4 weeks and the observed improvement is maintained at 12 months in the absence of significant adverse effects. It reduced tobacco craving, reactions to triggers, and compulsive behaviors typically linked to smoking, suggesting that it can also reduce the risk of relapse. Anti-NFL-101 IgG concentrations were higher in abstinent individuals.

Compared with current approved treatments for smoking cessation, NFL-101 has a favorable safety profile and the advantage of being administered only twice, an ease-of-use likely to improve adherence to treatment.

NFL-101 could potentially provide improved access to effective smoking cessation support to smokers who are unable to commit to frequent check-in visits, unable to remember to take daily medications or have limited access to well-established behavioral treatment programs.

Its efficacy on several key parameters including craving, coupled with a short treatment regimen, positions NFL-101 as a promising stand-alone or adjunct therapy. These findings support progressing to phase III, highlighting its potential as a new smoking cessation treatment.

## Supplementary Material

Supplementary_Table_1_ntaf181

Supplementary_Table_2_ntaf181

Supplementary_Table_3_ntaf181

## Data Availability

Data sharing is not possible because of Intellectual Property rights.
