# Supplementary material for: Efficacy and Safety of NFL-101 as a Smoking Cessation Therapy: A Randomized Phase II Clinical Trial CESTO2
Source: Nicotine Tob Res. 2025 Aug 30;28(4):586–94. doi: 10.1093/ntr/ntaf181 (PMC13008582; doi:10.1093/ntr/ntaf181)
Supplement: Supplementary_Table_1_ntaf181 [file supplementary_table_1_ntaf181.docx]

Supplementary Table 1: Treatment-emergent adverse events with a SOC incidence of at least 10% of subjects in either group

| System Organ Class / Preferred term | NFL-101 100 µg  N = 108 | NFL-101 200 µg  N = 109 | Placebo  N = 101 |
| --- | --- | --- | --- |
|  | N (%) | N (%) | N (%) |
| **General disorders & administration site condition** | **21 (19.4)** | **21 (19.3)** | **10 (9.9)** |
| Injection site pain | 7 (6.5) | 15 (13.8) | 4 (4.0) |
| Injection site reaction | 5 (4.6) | 3 (2.8) | 0 |
| Asthenia | 2 (1.9) | 2 (1.8) | 4 (4.0) |
| Fatigue | 1 (0.9) | 2 (1.8) | 2 (2.0) |
| Injection site erythema | 3 (2.8) | 2 (1.8) | 1 (1.0) |
| **Infections & infestations** | **26 (24.1)** | **25 (22.9)** | **18 (17.8)** |
| Covid 19 | 13 (12.0) | 9 (8.3) | 9 (8.9) |
| Bronchitis | 4 (3.7) | 5 (4.6) | 3 (3.0) |
| Influenza | 4 (3.7) | 5 (4.6) | 3 (3.0) |
| Nasopharyngitis | 2 (1.9) | 2 (1.8) | 3 (3.0) |
| **Nervous system disorders** | **13 (12.0)** | **17 (15.6)** | **6 (5.9)** |
| Headache | 6 (5.6) | 11 (10.1) | 3 (3.0) |
| Dizziness | 3 (2.8) | 1 (0.9) | 1 (1.0) |
| **Psychiatric disorders** | **15 (13.9)** | **9 (8.3)** | **12 (11.9)** |
| Anxiety | 1 (0.9) | 5 (4.6) | 4 (4.0) |
| Insomnia | 5 (4.6) | 5 (4.6) | 8 (7.9) |
| **Gastrointestinal disorders** | **12 (11.1)** | **5 (4.6)** | **13 (12.9)** |
| Nausea | 0 | 2 (1.8) | 4 (4.0) |
