# Supplementary material for: Efficacy and Safety of NFL-101 as a Smoking Cessation Therapy: A Randomized Phase II Clinical Trial CESTO2
Source: Nicotine Tob Res. 2025 Aug 30;28(4):586–94. doi: 10.1093/ntr/ntaf181 (PMC13008582; doi:10.1093/ntr/ntaf181)
Supplement: Supplementary_Table_2_ntaf181 [file supplementary_table_2_ntaf181.docx]

Supplementary Table 2: MMRM model comparing changes from baseline in anti-NFL-101 IgG between treatment arms

| **Groups comparison** | **Visit Day** | **LS Mean difference in anti-NFL-101 IgG** | **SEM** | **95% CI for LS Mean Difference** | **p-value** |
| --- | --- | --- | --- | --- | --- |
| 100-µg dose group -Placebo | 29 | 1.675 | 0.3410 | [1.004, 2.346] | <0.0001 |
|  | 43 | 1.855 | 0.3429 | [1.180, 2.530] | <0.0001 |
|  | 182 | 0.981 | 0.3457 | [0.300, 1.661] | 0.0049 |
| 200-µg dose group - Placebo | 29 | 2.864 | 0.3469 | [2.181, 3.547] | <0.0001 |
|  | 43 | 2.762 | 0.3492 | [2.075, 3.450] | <0.0001 |
|  | 182 | 1.779 | 0.3527 | [1.084, 2.473] | <0.0001 |
| 200-µg dose group - 100-µg dose group | 29 | 1.189 | 0.3398 | [0.521, 1.858] | 0.0005 |
|  | 43 | 0.907 | 0.3416 | [0.235, 1.580] | 0.0084 |
|  | 182 | 0.798 | 0.3418 | [0.125, 1.471] | 0.0203 |
