# Supplementary material for: Efficacy and Safety of NFL-101 as a Smoking Cessation Therapy: A Randomized Phase II Clinical Trial CESTO2
Source: Nicotine Tob Res. 2025 Aug 30;28(4):586–94. doi: 10.1093/ntr/ntaf181 (PMC13008582; doi:10.1093/ntr/ntaf181)
Supplement: Supplementary_Table_3_ntaf181 [file supplementary_table_3_ntaf181.docx]

Supplementary Table 3: Anti-NFL-101 IgG analyses

| **Parameters** | **Groups** | **Visit Day** | **LS Mean difference in anti-NFL-101 IgG between abstinents and non-abstinents** | **SEM** | **95% CI for LS Mean Difference** | **p-value** |
| --- | --- | --- | --- | --- | --- | --- |
| **ALL** | | | | | | |
| 4-week continuous abstinence confirmed by urinary cotinine | All | 29 | 1.274 | 0.3867 | [0.513, 2.035] | 0.0011 |
|  |  | 43 | 1.309 | 0.3854 | [0.550, 2.067] | 0.0008 |
|  |  | 182 | 0.958 | 0.3627 | [0.244, 1.672] | 0.0088 |
| 12-month continuous abstinence confirmed by urinary cotinine | All | 29 | 1.422 | 0.5033 | [0.432, 2.413] | 0.0051 |
|  |  | 43 | 1.520 | 0.5006 | [0.535, 2.506] | 0.0026 |
|  |  | 182 | 1.240 | 0.4652 | [0.324, 2.156] | 0.0082 |
| **Per arms** | | | | | | |
| 4-week continuous abstinence confirmed by urinary cotinine | Placebo | 29 | 0.269 | 0.6953 | [-1.100, 1.638] | 0.6989 |
|  |  | 43 | 0.150 | 0.6951 | [-1.219, 1.518] | 0.8297 |
|  |  | 182 | 0.072 | 0.6946 | [-1.295, 1.440] | 0.9170 |
|  | 100-µg dose group | 29 | 1.049 | 0.5292 | [0.007, 2.091] | 0.0484 |
|  |  | 43 | 0.983 | 0.5289 | [-0.058, 2.025] | 0.0640 |
|  |  | 182 | 0.507 | 0.5220 | [-0.521, 1.535] | 0.3323 |
|  | 200-µg dose group | 29 | 1.846 | 0.6105 | [0.644, 3.048] | 0.0027 |
|  |  | 43 | 2.066 | 0.6121 | [0.861, 3.271] | 0.0008 |
|  |  | 182 | 1.987 | 0.6030 | [0.799, 3.174] | 0.0011 |
| 12-month continuous abstinence confirmed by urinary cotinine | Placebo | 29 | 0.111 | 0.9173 | [-1.695, 1.917] | 0.9038 |
|  |  | 43 | 0.173 | 0.9174 | [-1.633, 1.979] | 0.8506 |
|  |  | 182 | 0.480 | 0.9122 | [-1.316, 2.276] | 0.5992 |
|  | 100-µg dose group | 29 | 1.548 | 0.6706 | [0.227, 2.868] | 0.0218 |
|  |  | 43 | 1.622 | 0.6705 | [0.302, 2.942] | 0.0162 |
|  |  | 182 | 0.979 | 0.6552 | [-0.311, 2.270] | 0.1362 |
|  | 200-µg dose group | 29 | 1.876 | 0.8177 | [0.267, 3.486] | 0.0225 |
|  |  | 43 | 1.958 | 0.8178 | [0.348, 3.568] | 0.0173 |
|  |  | 182 | 2.051 | 0.7978 | [0.480, 3.622] | 0.0107 |
